# Glucose starvation-mediated inhibition of salinomycin induced autophagy amplifies cancer cell specific cell death

**DOI:** 10.18632/oncotarget.3548

**Published:** 2015-03-12

**Authors:** Jaganmohan R. Jangamreddy, Mayur V. Jain, Anna-Lotta Hallbeck, Karin Roberg, Kourosh Lotfi, Marek J. Łos

**Affiliations:** ^1^ Department of Clinical & Experimental Medicine (IKE), Division of Cell Biology, Integrative Regenerative Medical Center (IGEN), Linköping University, Linköping, Sweden; ^2^ Department of Clinical and Experimental Medicine, Division of Oncology, Linköping University, County Council of Östergötland, Linköping, Sweden; ^3^ Division of Oto-Rhino-Laryngology and Head and Neck Surgery, Department of Clinical and Experimental Medicine, Faculty of Health Sciences, Linköping University, Linköping, Sweden; ^4^ Clinical Pharmacology, Division of Drug Research, Department of Medical and Health Sciences, Linköping University, Linköping, Sweden; ^5^ Department of Hematology, County Council of Östergötland, Linköping, Sweden; ^6^ Department of Pathology, Pomeranian Medical University, Szczecin, Poland

**Keywords:** glucose starvation, 2DG, 2FDG, normoxia, hypoxia

## Abstract

Salinomycin has been used as treatment for malignant tumors in a small number of humans, causing far less side effects than standard chemotherapy. Several studies show that Salinomycin targets cancer-initiating cells (cancer stem cells, or CSC) resistant to conventional therapies. Numerous studies show that Salinomycin not only reduces tumor volume, but also decreases tumor recurrence when used as an adjuvant to standard treatments. In this study we show that starvation triggered different stress responses in cancer cells and primary normal cells, which further improved the preferential targeting of cancer cells by Salinomycin. Our *in vitro* studies further demonstrate that the combined use of 2-Fluoro 2-deoxy D-glucose, or 2-deoxy D-glucose with Salinomycin is lethal in cancer cells while the use of Oxamate does not improve cell death-inducing properties of Salinomycin. Furthermore, we show that treatment of cancer cells with Salinomycin under starvation conditions not only increases the apoptotic caspase activity, but also diminishes the protective autophagy normally triggered by the treatment with Salinomycin alone. Thus, this study underlines the potential use of Salinomycin as a cancer treatment, possibly in combination with short-term starvation or starvation-mimicking pharmacologic intervention.

## INTRODUCTION

Initially proposed in 1930's, Warburg effect or the dependence of cancer cells on aerobic glycolysis, is considered the ‘Achilles heel’ of cancer [1]. The addiction of cancer cells to accumulate the cellular mass increases uptake of glucose as opposed to normal cells that undergo quiescence/senescence under nutrient deprivation, even in the presence of growth factors. This adoption of proliferative cancer cells for survival can be exploited for preferential targeting [1].

Even though current treatment procedures are able to effectively target the bulk of the tumor, cancer recurrence and metastasis formation are major reasons leading to therapy failure. Studies over the last decade show that the drug-resistant cancer initiating cells (cancer stem cells, CSC) have similar characteristics to stem cells as far as self-renewal and to some extent also differentiation capacities [2-4]. In 2009, Gupta and colleagues screened about 16000 compounds in the quest to identify molecules that are preferentially toxic to CSC. The screen identified, an antibiotic with K^+^-ionophore properties Salinomycin, which has been used for decades in animal farming for both increasing nutrient absorption and treatment for parasitic infections (e.g. coccidiosis) [5].

Consistent with these findings, the effective targeting of CSC by Salinomycin in several malignancies including breast-, prostrate-, brain-, blood-, liver-, pancreatic-, and lung cancers was further established [6-11]. Salinomycin kills cancer cells by a mixed apoptotic and autophagic form of cell death, while the latter one is initially induced as a protective mechanism [9, 12-14]. So far, lethal toxicity of Salinomycin to humans was not reported. One case of accidental high dose exposure to Salinomycin of a farm-worker has been documented [15]. Using *in vitro-*studies, Boehmerle and colleagues showed that Salinomycin is toxic to normal neuronal cells (murine dorsal root ganglion neurons, toxicity at 1μM, cell viability ~25%, *in vitro*-experiment), and thus is expected to cause mild to severe neuropathies [16]. More recently, the work from the same group, using mouse models, show that a combination of Salinomycin (5mg/kg daily injection), with inhibition of mitochondrial Na^+^/K^+^ exchanger was able to show no such neuronal toxicity, without altering the cancer cell cytotoxicity [17]. Furthermore, partially successful pilot study in humans showed minor secondary symptoms while causing the regression of metastatic tumor [6]. Thus, the efficacy of Salinomycin will likely be further clinically tested among wide range of cancer patients [6].

Salinomycin's ability to specifically kill slowly proliferating cancer stem-like cells more robustly than the differentiated cancer cells, even at lower concentrations, lead to studies using commonly used chemotherapeutic agents in combination with Salinomycin [6, 18, 19]. We have previously observed that salimomycin caused mitochondrial dysfunction, decrease of cellular ATP, and induction of autophagy [9, 14]. Thus, following on our previous findings, in this study, we tested the response of normal- and cancer cells under starvation conditions (natural autophagy inducer). We studied Salinomycin's toxicity under glucose starvation, or under competitive inhibition of glycolytic pathway (pharmacological triggered starvation-like conditions), as well as under hypoxia (natural inhibition of phosphorylative oxiation).

## RESULTS

### The kinetics of Salinomycin-induced cell death

**Figure 1:**
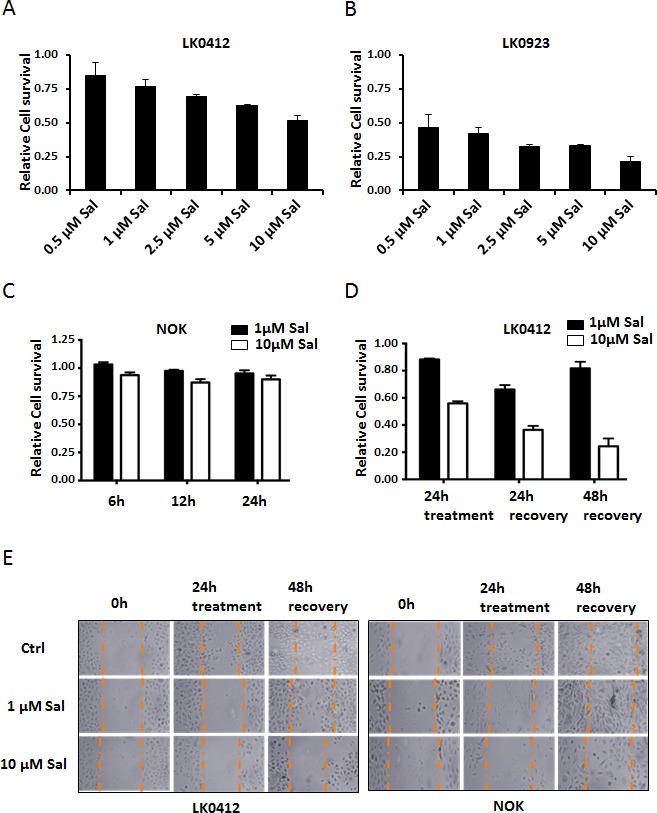
The kinetics of Salinomycin-induced cell death (A and B) MTT assay was employed to assess cell viability upon cell treatment with various concentrations of Salinomycin for 24h. (C) Primary NOK cells, treated with 1μM and 10μM Salinomycin for up to 24h were resistant to Salinomycin. (D) LK0412 cells were pretreated with 10μM Salinomycin for 24h, then medium was replaced with normal keratinocyte media for 48h, and then cells viability were assessed by MTT-assay. (E) Scratch was made with pipette tip, among fully confluent NOK and LK0412 cells cultured in 3 cm Petrie dishes. Cells were then treated with 1μM and 10μM Salinomycin for 24h. Salinomycin was removed by medium replacement, and 48h later cell proliferation into the scarred area was assessed microscopically and documented using JuLi microscope, (N=3).

Salinomycin efficiently kills a variety of cancer cells, however it spares normal primary cells (primary human dermal fibroblasts and primary human hepatocytes) at least within the tested therapeutic window [9, 20]. As shown in Fig. 1A and 1B, Salinomycin kills the tongue and larynx cancer cell lines LK0412 and LK0923 respectively, in a concentration-dependent manner. Interestingly, LK0923 cells that have a high percentage of cells expressing CD44 show a higher toxicity by Salinomycin than LK0412. However, normal oral keratinocytes (NOK) did not show any significant cell death after 24h of treatment with 1μM and 10μM Salinomycin (Fig. 1C). We further studied the reversibility of cell death and effect on cell proliferation by Salinomycin treatment. MTT assay results showed that LK0412 cells treated with 10μM Salinomycin for 24h, after which the medium was replaced with Salinomycin-free media for another 48h, did not show increase in cell proliferation but instead further decrease in cells viability (Fig. 1D). Such changes may indicate Salinomycin's preferential toxicity towards residual CSC. Following-up these results, we conducted ‘wound-healing’ assay among both cancer cells (LK0412) and NOK. Cancer cells, treated with 1μM Salinomycin, showed partial recovery during the 48h post-treatment period after media have been changed. However when 10μM Salinomycin was applied, no signs of recovery could be observed in cancer cells (Fig. 1E). In contrast, NOK cells showed recovery at both concentrations (1μM and 10μM), even signs of hypertrophy at 1μM concentration of Salinomycin (Fig. 1E).

### Glucose starvation and Salinomycin synergistically kill cancer cells while protecting normal, primary cells

**Figure 2:**
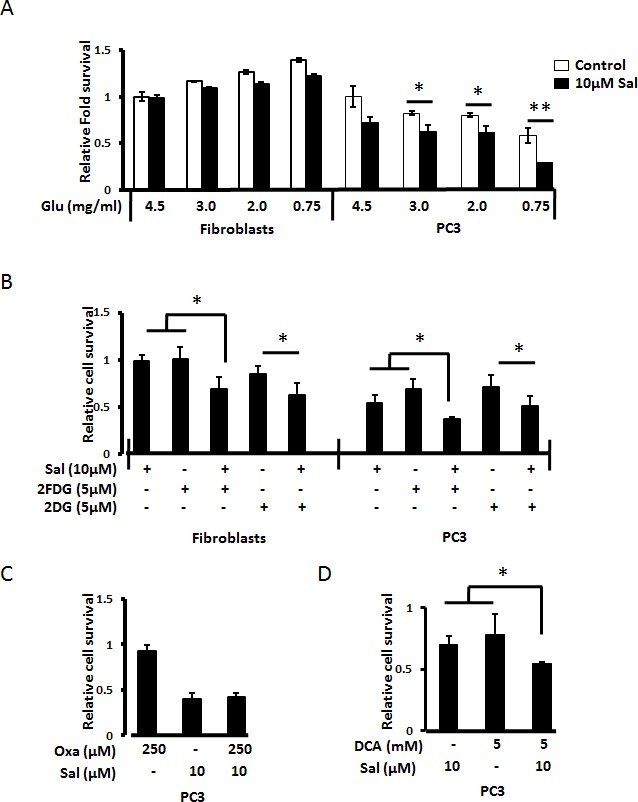
Starvation potentiates Salinomycin's preferential toxicity towards cancer cells (A) Human dermal primary fibroblasts and PC3 prostate cancer cells grown to 100% confluence, as described in methods section show differential stress response to Salinomycin under serum (1% FBS) and glucose-starved conditions. Cell survival was assessed by MTT-assay. PC3 cells were readily dying (~15% survival rate after 24h) upon Salinomycin treatment under afore-mentioned conditions, while primary normal fibroblasts survived under the same conditions. (B) Glucose-starved was pharmacologically simulated by using competitive glucose transport inhibitors 2FDG and 2DG. Salinomycin treatment in combination with 5μM 2FDG and 5μM 2DG show a profound cell death in PC3 cells while primary normal cells did show only minimal toxicity under such conditions. However, (C) inhibition of lactate dehydrogenase with sodium oxamate (pretreatment for 1h) did not potentiate Salinomycin's toxicity even after 72h of treatment, but (D) inhibition of pyruvate dehydrogenase with DCA shows increase in cell death by salinomycin at 48h (N=3, *p<0.05, **p<0.01).

To further enhance Salinomycin induced toxicity among cancer cells along with rendering protection to the primary cells we looked if the ‘Warburg effect’-dependent cancer cell behavior could affect Salinomycin's preferential toxicity towards cancer cells. When treated with Salinomycin under the conditions of glucose starvation and 1% FBS, Salinomycin induced cell death increased three-fold in PC3 cancer cells (Fig. 2A). However primary human fibroblasts showed increased cell survival under the same conditions (Fig. 2A). We have tested glucose levels up to 0.75mg/ml, as such levels may be achieved in patient's tissues upon starvation. Glucose analogues 2DG and 2FDG employed in combination with Salinomycin (pharmacologically-induced glucose starvation), similarly increased Salinomycin's toxicity in PC3 cells, but they were also partially toxic towards normal primary fibroblasts (Fig. 2B).

Since, lack of glucose or its competitive inhibitors (2DG and 2FDG) attenuates glycolysis and thus inhibiting the main source of energy among cancer cells, we next checked how the inhibition of conversion of pyruvate to lactate, using sodium oxamate, affects salinomycin's toxicity. Surprisingly, the inhibition of the conversion of pyruvate to lactate by sodium oxamate, in PC3 cells, had no effect on Salinomycin's toxicity, even at 10μM concentration, and after 72h (Fig. 2C). So, we further followed the inhibition of oxidative phosphorylation with dichloroacetate (DCA) that activates pyruvate dehydrogenase by inhibition of pyruvate dehydrogenase kinase. PC3 cells that are pretreated with 5mM DCA for 1 h and then treated with salinomycin showed increased cell death compared to individual treatments of 5mM DCA and 10μM Salinomycin (Fig. 2D).

### Hypoxia promotes Salinomycin-induced cell death

**Figure 3:**
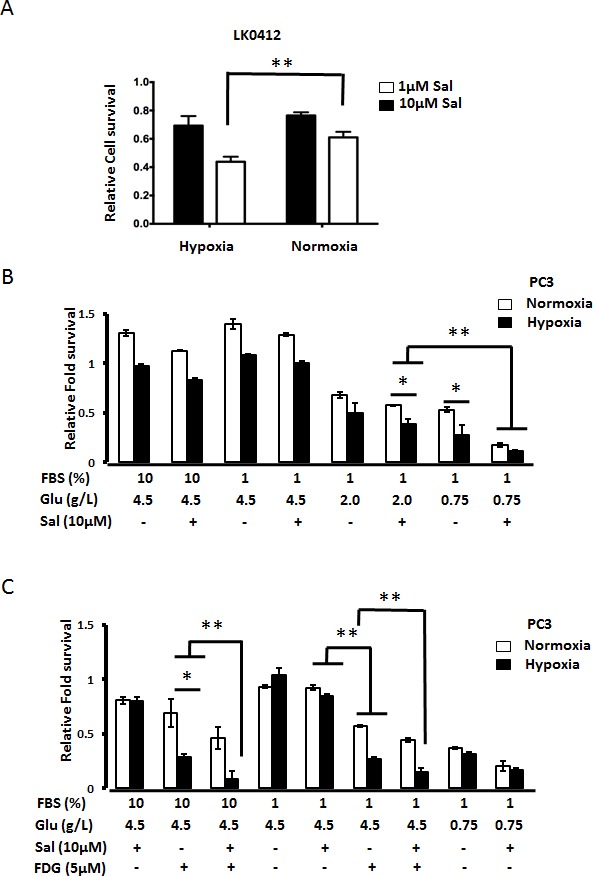
Hypoxia potentiates Salinomycin-triggered cell death (A) Hypoxic conditions amplify Salinomycin's toxicity against cancer cells. LK0412 cells were tested for Salinomycin's toxicity under normoxia or under hypoxia (1% of oxygen). 24h later cell viability was assessed by MTT assay. Similarly, (B) PC3 cells were tested for Salinomycin's toxicity, under combination of factors like hypoxia, normoxia, serum starvation and various glucose levels in the medium, referred to in the text as stressors. 24h later cell viability was assessed by MTT assay. (C) In a similar as in “B” experimental setup, in addition to glucose starvation, competitive inhibitor of glycolysis, 2FDG was employed through the duration of the experiment to pharmacologically mimic glucose starvation. (N=3, *p<0.05, **p<0.01).

CSCs survive well under hypoxic conditions found within hypo-perfused parts of a tumor. Furthermore, hypoxic conditions, and decreased drug-bioavailability promote drug resistance, thus we have tested how hypoxic conditions affect Salinomycin's toxicity. As shown in Fig. 3A and 3B, Salinomycin efficiently kills both LKO412 and PC3 cells under hypoxic conditions. We have next tested how glucose starvation affects Salinomycin's toxicity under hypoxic conditions. As shown in Fig. 3B, Salinomycin triggered cell death in PC3 cells, also under low glucose conditions. A repetition of experiments in the presence of glucose analogue 2FDG, produced comparable effects among cancer cells, within 24h of treatment (Fig. 3C). The tests were performed under both 10% and 1% FBS concentrations to probe different conditions within tumor.

### Glucose starvation inhibits Salinomycin induced autophagy

**Figure 4:**
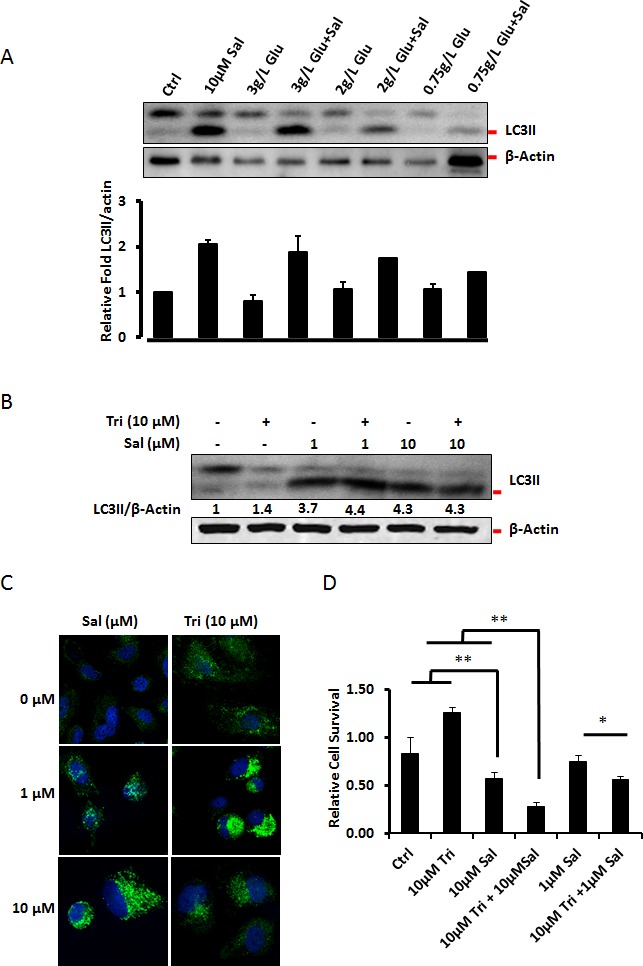
Starvation inhibits autophagic response to Salinomycin (A) Salinomycin triggered autophagy was attenuated under glucose starvation conditions. Cells were cultured to full confluence and treated as for the studies of differential stress response and autophagy was monitored by Western blot detection of LC3II form. The Western blot membranes were scanned and quantified densitometrically. Lower panel shows ratiometric changes of LC3II form expressed as a fold increase compared to control. ß-actin, was used as a loading control, and for normalization purposes. (B, C) Inhibition of Akt signaling pathway by Triciribine, mimics serum starvation by inactivating downstream signaling cascade involving mTORC1, and promotes autophagy. The effect of Akt inhibition on Salinomycin-induced autophagy was (B) assessed by Western blot detection of LC3II levels, and (C) detection of LC3 punctae by Immuno-cytochemistry. Triciribine treated cells and Salinomycin (1μM and 10μM) treated cells show increase in LC3II levels with Western blots and increased LC3 punctae as observed in the representative images, independently. (D) The effects of Akt-inhibition by Triciribine on Salinomycin's toxicity were assessed by MTT assay, after 48h of treatment. Salinomycin toxicity increased synergistically in the presence of Triciribine, which alone was not toxic (N=3, *p<0.05, **p<0.01).

Our earlier studies show that cells respond to Salinomycin treatment with autophagy, as a cell survival mechanism. However to our surprise, under glucose starvation conditions, Salinomycin treatment did not show increased LC3II accumulation but rather showed dramatic decrease, and thus implying a marked decrease in autophagy among extremely stressed cells (Fig. 4A, and Suppl. Fig. 1). Akt signaling increases if nutritents are readily available whereas starvation downregulates Akt-activity and induces autophagy. Thus, we mimicked this aspect of starvation-related event using Akt inhibitor, and studied the effect of Salinomycin under Akt compromised conditions. As shown in Fig. 4B treatment of 1μM Salinomycin in the cells pre-treated with the Akt inhibitor Triciribine, triggered increased accumulation of the autophagic marker LC3II. However, under the same conditions in the presence of 10μM Salinomycin we did not observe any change in LC3II levels compared to Salinomycin treatment alone (Fig. 4B). Similar observations were made using immuno-cytochemistry and confocal microscopy (Fig. 4C). We next checked how Triciribine affects Salinomycin's toxicity. As shown in Fig. 4D, Triciribine potentiated Salinomycin's toxicity, both at 1μM and 10μM concentrations. Interestingly, combination of 1μM Salinomycin with 10μM Triciribine caused similar level of toxicity in PC3 cells as 10μM Salinomycin alone.

### Glucose starvation activates caspase-dependent form of cell death (apoptosis) by Salinomycin

**Figure 5:**
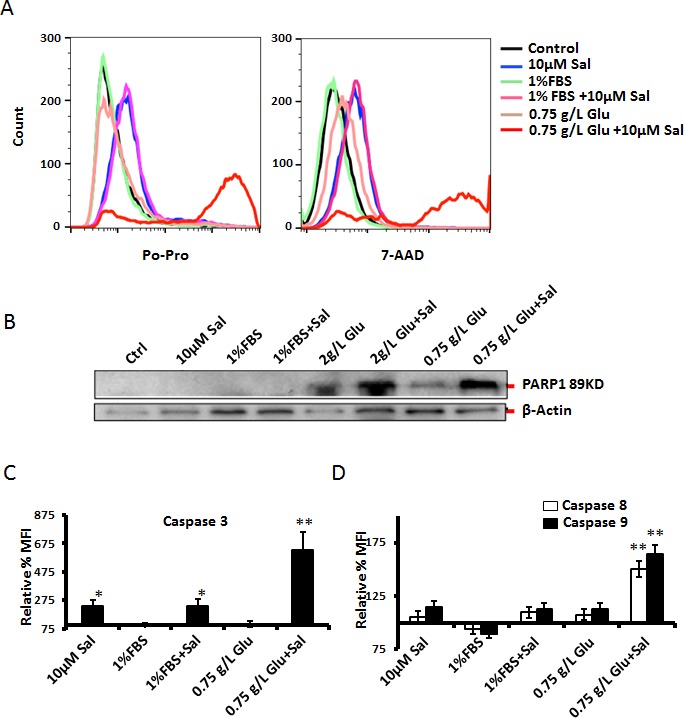
Assessment of cell death mechanism triggered by Salinomycin – effects of starvation (A) PC3 cells were grown to reach full confluency for 48h, using normal media (10% FBS and 4.5 g/L glucose). The cell culture medium for control cells was replaced with normal medium, where as the experimental samples were provided with media containing 1% FBS and 0.75 g/L glucose, after washing with PBS. Salinomycin was added to experimental samples 1h after respective media replacements, and 24h-incubation followed. The forms of cell death, and the effects of serum- or glucose starvation on Salinomycin's toxicity were assessed by flow cytometry. Apoptosis was detected by Po-Pro staining, and necrosis was assessed by 7AAD-staining. (B) To confirm that apoptosis may have been induced by Salinomycin under starvation conditions PARP1-cleavage (indicator of active caspase-3 or -7) was detected by Western blotting in cells prestarved as in “A”, and then treated with Salinomycin for 24h. (C and D) To check which caspases were involved in Salinomycin-induced cell death, under normal, or starvation conditions, the activity of caspases-3, -8 and -9 was assessed by flow cytometry, in a similar experimental settings as in “A”, (N=3, *p<0.05, **p<0.01). Control sample were non-treated cells but handled and stained similarly to experimental samples. The control ‘mean fluorescence intensity’ (MFI) was considered as 100% and a relative change in MFI among test samples was calculated accordingly.

In our initial experiments we have used MTT assay, which only assesses cell survival, cell proliferation, but cannot distinguish between apoptosis and necrosis. To assess the form of cell death initiated by Salinomycin under tested experimental conditions, we have employed the flow-cytometry-based Po-Pro/7-AAD assay. High Po-Pro staining is indicative for apoptosis, whereas high 7-AAD signal indicates compromised cell membranes and necrosis; also late apoptotic cells stain with for both dyes. Under glucose starvation, and in the presence of Salinomycin, dying cells stained with both Po-Pro and 7-AAD, thus indicating apoptosis induction under such experimental conditions (Fig. 5A). To further elucidate the molecular mechanism of cell death under combined starvation and Salinomycin treatment, we checked for signs of executioner-caspase-3 (or -7) activity by using the cleavage of PARP1 as indicator (Fig. 5B). Indeed, Salinomycin treatments amplified glucose starvation-triggered PARP1-cleavage. To gain more insight into the molecular mechanism of cell death, under tested experimental conditions, we directly measured the activity of caspases-3, -8, and -9 by flow cytometry. Combination of glucose starvation and Salinomycin potentiated the activation of all three caspases tested, thus confirming that the combination of starvation and Salinomycin induces caspase-dependent cell death (Fig. 5C and 5D).

## DISCUSSION

Salinomycin gained substantial attention when it was identified as a drug preferentially killing breast cancer cells exhibiting stemness characteristics [5]. Salinomycin induced robust cell death in E-cadherin-negative cells (characteristic typical for metastasis-forming cells) as compared to the respective control counterparts [5]. Hence, Salinomycin's anticancer properties have been evaluated in combination with conventional anticancer drugs. Interestingly, Salinomycin kills Doxorubicin-, Cisplatin-, Gemcitabine-, Temozolamide-, Tratsuzumab- and Imatinib-resistant cells, and counteracts tumor recurrence in *in vitro* as well as animal models [6, 18, 21-23]. Interestingly, in this study, we show that LK0923 cells that express higher level of CD44 than LK0412 cells are more susceptible to Salinomycin (Fig. 1A and 1B) [24].

Classical radio- or chemotherapy leads to the selection of the therapy-resistant clones that cause the recurrence of malignant disease [25, 26]. Our study, employing wound healing assay and MTT assay show that Salinomycin treatment specifically inhibits the proliferation of cancer cells, following treatment, without the mergence of clones that could repopulate the killed cells, or the “scratch area”. Interestingly, no such inhibition of proliferation was observed among corresponding primary NOK, even though we occasionally observed an increase in cell size. Hence, the data indicates that Salinomycin preferentially targets CSC without causing major alteration to the primary cells.

Another factor affecting the action of anticancer drugs is the tumor microenvironment [3]. Parts of tumor may be deprived of oxygen (hypoxia) along with accumulation of metabolites of glycolysis that decrease the pH and may influence pharmaco-kinetics of drugs. Our data indicate that both hypoxia and starvation conditions amplify Salinomycin's action. Salinomycin has been killing cancer cells more efficiently under hypoxic conditions rather than normoxic conditions. Drawing inspiration from previous work on differential stress response (DSR) by normal primary and cancer cells, we tested Salinomycin's toxicity under low glucose and low serum exposure at levels achievable upon starvation [27, 28]. Salinomycin's toxicity was strongly potentiated in cancer cells, at glucose levels achievable by starvation (0.75 g/L), and under low serum supply (1% FBS), while primary human fibroblasts were resistant to Salinomycin. Our previous studies show that, among other effects, Salinomycin triggers cell death through damage to mitochondria leading to decrease of cellular ATP level [9, 14]. Thus, when Salinomycin acts under low glucose level (the primary energy source for cancer cells), its toxicity towards cancer cells will be strongly amplified. Importantly, increased Salinomycin's specificity towards cancer cells under starvation condition was further enhanced under hypoxia. Similarly, glucose starvation mimicked by using glucose analogues that cannot enter glycolysis pathway, also potentiated Salinomycin's toxicity both under normoxic and hypoxic conditions, irrespective of serum content (Fig. 3). However, Salinomycin in the presence of glucose analogues was partially toxic towards normal primary fibroblasts (Fig. 2B). The above experiments show that Salinomycin is more effective under conditions mimicking intra-tumor environment, and that natural starvation, rather than pharmacologic inhibition of glucose uptake, would be potentially more favorable conditions to potentiate therapeutic effect of Salinomycin.

While combination of treatment with glucose analogues (2DG, 2FDG) potentiated Salinomycin's toxicity, co-treatment with sodium oxamate that inhibits formation of Lactate (late stage of anaerobic glycolysis in human cells) did not. This observation further underlines the dependence of cancer cells on glycolysis-derived ATP. Our further studies using DCA, which inhibits pyruvate dehydrogenase kinase resulting in the activation of mitochondrial pyruvate dehydrogenase complex that catalyzed the conversion of pyruvate formed at the end of glycolysis stem to acetyl-CoA molecules that enter TCA cycle, in combination with salinomycin show an increase in cell death. These results suggest that the promotion of oxidative phosphorylation further potentiates salinomycin induced cell death. DCA is previously shown to initiate mitochondrial dependence of cancer cells for ATP production through normalization of dysfunctional mitochondria and there by activating intrinsic cell death pathway in cancer cells [29, 30]. On the other hand, salinomycin being an ionophore with specificity for K^+^ ions promote hyperpolarization of mitochondria or erythrocytes but usage of higher concentrations show no such specificity resulting in net depolarization [31, 32]. These effects on mitochondrial polarity leading to altered metabolic dependence of cancer cells could be the reason for additive cell death effect of DCA and salinomycin among cancer cells.

We have previously shown that Salinomycin triggers cell death through interference with mitochondrial ATP production that strongly induces protective autophagy. Consequently, the metabolic disturbances trigger caspase activity and apoptotic cell death [9, 14]. Consistently with the results reported by us, and by other labs [6, 9], starvation conditions, which may lower ATP-level, in combination with Salinomycin, trigger caspase-3, -8 and -9 activity and apoptotic cell death. More over salinomycin is shown to activate the AMP activated protein kinase (AMPK) that triggers autophagy and similarly starvation conditions or the mimicking conditions promote AMPK activity [33, 34]. This synergistic activation of AMPK activity by salinomycin as well as starvation conditions could cause the inhibition of cell proliferation and promote cell death.

The activation of autophagy by Salinomycin modulates its toxicity [9, 35]. We have also shown that serum starvation potentiates Salinomycin's toxicity. Serum starvation also affects the pro-survival Akt-kinase activity. We next tested how triciribine, the inhibitor of pan-Akt-kinases, affects Salinomycin's toxicity. Triciribine strongly potentiated Salinomycin's toxicity, which confirms our previous results that the decrease of pro-survival signals (serum starvation) potentiates Salinomycin's toxicity.

In conclusion, we show that hypoxic, low-glucose, low-nutrient and growth factor conditions found within hypo-perfused tumor, actually potentiate Salinomycin's toxicity, and increase its preferential anticancer activity. While starvation, that amplifies selective antitumor activity of Salinomycin, could be pharmacologically mimicked by the application of glucose analogues (2DG, 2FDG), likely ordinary fasting is a clinically-safer method to potentiate Salinomycin's action, and increase its therapeutic window. It would be interesting to test salinomycin in combination with biologics that selectively kill cancer cells, like i.e. apoptins [36-38], to see possible synergy effects.

## MATERIALS AND METHODS

### Cells and cell culture

PC3 (Human prostrate cancer cell line) and human primary dermal fibroblasts cells were cultured in complete media (RPMI and DMEM media respectively, supplemented with 10% FBS and 1% penicillin-streptomycin antibiotics) and maintained at sub-confluent conditions as described previously [9]. LK0412 and LK0923 (tongue and larynx cancer cells respectively) and NOK (Normal Oral Keratinocytes) were cultured in Keratinocyte-SFM (GIBCO, Invitrogen Corporation, Paisley, UK) supplemented with antibiotics (penicillin 50 IU/ml, streptomycin 50 μg/ml) [39].

### Materials and reagents

Salinomycin, 2DG, 2FDG and Saponin, were obtained from Sigma-Aldrich and dissolved in their respective buffers as per required concentrations. Antibodies were obtained from the following sources: Rabbit-anti-LC3b (Sigma Aldrich), murine anti-Actin (Abcam) Rabbit anti-PARP1 cleaved subunit antibody (Millipore). Secondary antibodies anti-rabbit HRP-conjugate and anti-mouse HRP-conjugates were obtained from Sigma-Aldrich.

### Cell death and cell proliferation assays

MTT assay was conducted to assess cell survival and cell proliferation as described in our earlier studies [9, 40]. 10,000 cells plated in each well of a 96 well plate 24h prior to experimental treatments. After mentioned experimental treatments, cells were incubated with MTT solution for 4h, the formazan crystals were dissolved in DMSO:Ethanol (1:1 ratio), and the readings were obtained using a spectrophotometer as described previously [9]. For differential stress response studies, cells were grown to a confluence (48h), then the media were replaced with low glucose and/or FBS contents-media, for 6h and subsequently treated with Salinomycin for 24h before harvesting for analysis.

### Assessment of cell death and caspase activity by flow cytometry

Cells were plated in a 6 well plate at 150,000 cells/well and were cultured to full confluence (48h) prior to treatment procedures, as indicated in the respective experimental setup. After 24h of treatment, under various conditions of glucose and serum levels, with or without the presence of Salinomycin, the cells were trypsinized, centrifuged and resuspended in 500μl of PBS. The cells were then stained with Po-Pro and 7AAD (Life technologies Ltd.) by incubating on ice for 30 min, and were measured by flow cytometry as described previously [9]. To assess the activity of caspase-3, -8 and -9, the cells were collected in a similar way as for Po-Pro and 7AAD assay, and were incubated with FAM-FLICA caspase-3, FAM-FLICA caspase-8, and SR-FLICA caspase-9 substrates (ImmunoChemistry Technologies) for 30 min, on ice in individual aliquots. The caspase activities were then measured by flow cytometry, as described in detail previously [9, 41].

### Immunoblotting

For Western blot analysis collected cell lysates were assessed for protein concentration using Bradford assay and further handled as described previously [37]. 20μg of protein was loaded per gel-well.

### Confocal microscopy

Before imaging using confocal microscope (Zeiss), cells were fixed and permeabilized with 4% paraformaldehyde and methanol, respectively. Cells were then stained for LC3, using anti-LC3b antibody obtained from Sigma-Aldrich, overnight in incubation buffer (0.1% Saponin and 5% FBS in PBS). Next day, cells were washed with incubation buffer (3 times, 5 min each wash) and were incubated with alexa-488 conjugated anti-Rabbit antibody (Life Technologies Ltd) for 1h. The cells were washed and mounted before imaging. A detailed description of staining procedure was published previously [36, 42].

### Wound healing assay

A straight line (wound) was made in between the 80% confluent cells grown in a 6-well plate with a sterile pipette tip and treated with Salinomycin for respective time periods. Images were taken after 48h using a JuLi Smart Fluorescence cell Imager in the bright field.

### Statistics

All the statistics (one way ANOVA, and two way ANOVA) were conducted using Prism (version 6.0b) and SPSS (IBM version 20) softwares. All the experiments were represented as means of a minimum of 3 independent experiments unless otherwise mentioned. A p value of less than 0.05 was considered statistically significant, unless mentioned otherwise.

## SUPPLEMENTARY MATERIAL, FIGURE



## Abbreviations

2DG: 2-deoxy d-glucose
2FDG: 2-Fluoro deoxy d-glucose
7-AAD: 7-Aminoactinomycin D
CSC: cancer-initiating cells (cancer stem cells)
DSR: Differential stress response
NOK: Normal Oral Keratinocytes
Oxa: Sodium Oxamate
PARP1: Poly (ADP-ribose) polymerase 1
Sal: Salinomycin.
