# Supplementary material for: Glucose starvation-mediated inhibition of salinomycin induced autophagy amplifies cancer cell specific cell death
Source: Oncotarget. 2015 Mar 12;6(12):10134–45. doi: 10.18632/oncotarget.3548 (PMC4496345; doi:10.18632/oncotarget.3548)
Supplement: Supplementary file 1 [file oncotarget-06-10134-s001.pdf]

Glucose starvation-mediated inhibition of salinomycin induced autophagy amplifies cancer cell specific cell death

Supplementary Material

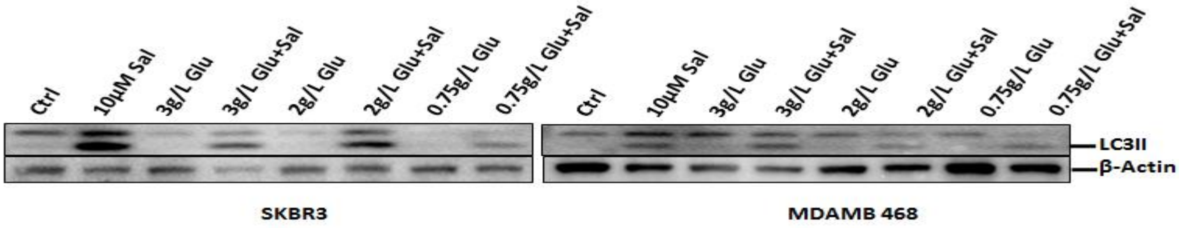

Supplementary Figure 1: Inhibition of Salinomycin-induced autophagy by starvation is cancer cell type independent: In a similar experimental settings as for experiments shown in figure 4, where PC3 cells were tested, we further checked the LC3II-form quantity upon Salinomycin treatment, combined with glucose- and FBS-starvation, in breast cancer cells SKBR3 and MDAMB468. The data obtained in these cell line models confirmed results obtained in PC3 cells.
